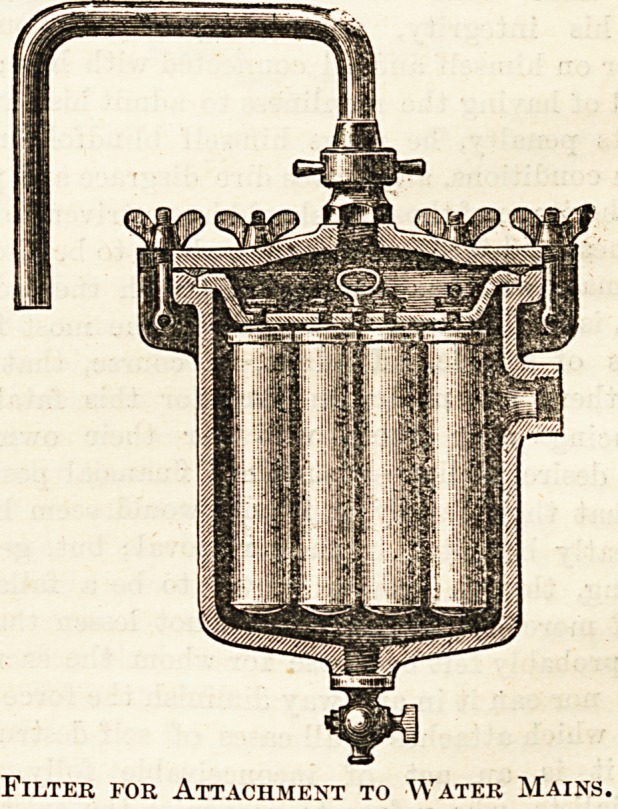# New Appliances and Things Medical

**Published:** 1899-09-23

**Authors:** 


### NEW APPLIANCES AND THINGS MEDICAL.

[We shall be glad to receive, at our Office, 28 & 29, Southampton Street, Strand, London, W.C., from the manufacturers, specimens of all new preparations and appliances which may be brought out from time to time.]

### TANNOFORM.

(E. Merck, Darmstadt.)

This new body is a condensation product of tannin and formaldehyde of the formula $C_{29} H_{20} O_{18}$. It is a reddish-white powder, insoluble in water, but soluble in alcohol and most alkalis. Its chief application is as a desiccative antiseptic, and as such is particularly valuable in the treatment of weak ulcers, bed sores, and other conditions of macerated epidermis; up to the present time it has been chiefly tried in cases of hyperidrosis and bromidrosis, and when this condition affects the parts between the toes, and is accompanied with a moist and macerated condition of the skin, it may fairly be regarded as a specific. The astringent and antiseptic properties of the drug individually exercise a most healthful action on the condition, and in such cases as we have had opportunity of trial the reports have been most satisfactory. It is regarded as a most agreeable application, being odourless and non-irritating, and at the same time rapid in its action. As compared with the older method of applying tannic acid, it is superior in every point, and we strongly recommend practitioners to make a note of this new remedial application for all conditions of hyperidrosis or offensive perspiration.

### THE IDEAL FILTER.

(Richard Simon, Vernon Road, Nottingham.)

This filter, which has the widest possible range of applications, from the pocket travelling size to such as is required for town or municipal supplies, consists of a single porous tube, or a number of such tubes. The tubes are made of artificial insoluble stone, and the water passes from the outside to the inside, the impurities being deposited on the former surface, whence they can be easily and effectually removed by occasional washing, which is done without removing the tubes or the frame in which they are suspended, by simply a flushing cock. The ideal filter can be fitted to supply-pipes, the water being then filtered under pressure, or where there is no such supply it can be adapted to a force pump, which in a modified form is invaluable for military purposes. The domestic filter, of which we give an illustration, is recommended for large households, hotels,

The Ideal Filter.

schools, institutions, &c. It is made of zinc, thoroughly enamelled inside and outside, and furnished with wrought-iron stand. The cylinder containing the filter tubes has also a chamber for ice. The rate of filtration is about two gallons per hour, and the price is £4 10s. The other illustration which we append is a water-supply filter to be attached to mains, with a pressure of about 32 feet. The water thus

Filter for Attachment to Water Mains.

filtered can be forced out some height above the level of the filter. The discharge cock at the bottom serves to remove the impurities deposited. Over 100 gallons of water per hour can be filtered by this means, and the price is £4.

## Figures and Tables

**Figure f1:**
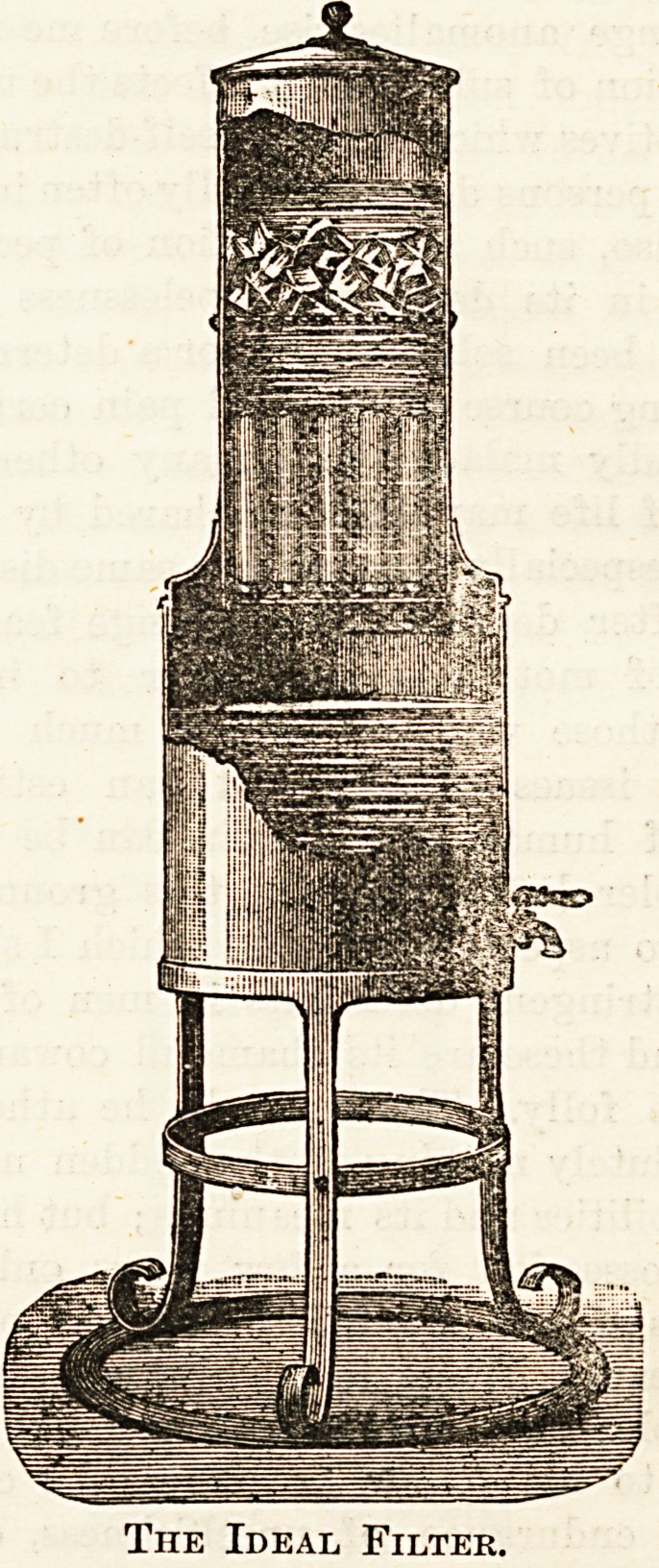


**Figure f2:**